# Insight into functionalized-macrocycles-guided supramolecular photocatalysis

**DOI:** 10.3762/bjoc.17.15

**Published:** 2021-01-18

**Authors:** Minzan Zuo, Krishnasamy Velmurugan, Kaiya Wang, Xueqi Tian, Xiao-Yu Hu

**Affiliations:** 1 College of Materials Science and Technology, Nanjing University of Aeronautics and Astronautics, Nanjing, 211106, China

**Keywords:** host–guest chemistry, macrocycles, noncovalent interactions, supramolecular photocatalysis

## Abstract

Due to the unique characteristics of macrocycles (e.g., the ease of modification, hydrophobic cavities, and specific guest recognition), they can provide a suitable environment to realize photocatalysis via noncovalent interactions with different substrates. In this minireview, we emphasized the photochemical transformation and catalytic reactivity of different guests based on the binding with various macrocyclic hosts as well as on the role of macrocyclic-hosts-assisted hybrid materials in energy transfer. To keep the clarity of this review, the macrocycles are categorized into the most commonly used supramolecular hosts, including crown ethers, cyclodextrins, cucurbiturils, calixarenes, and pillararenes. This minireview not only summarizes the role that macrocycles play in photocatalytic reactions but also clarifies the photocatalytic mechanisms. Finally, the future research efforts and new pathways to apply macrocycles and supramolecular hybrid materials in photocatalysis are also discussed.

## Introduction

**Figure 1:**
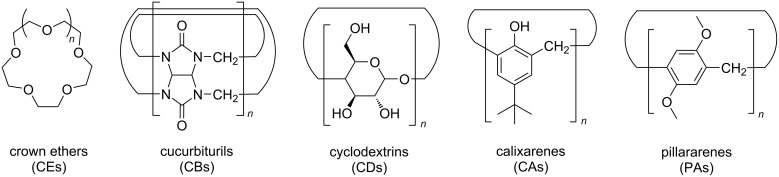
Chemical structures of representative macrocycles.

Enzyme-catalyzed reactions are often carried out fantastically in nature via noncovalent interactions of a substrate [1–2]. Inspired by these natural processes, chemists have begun to develop artificial supramolecular systems that provide a similar environment to perform various catalytic reactions where substrates are captured via host–guest interactions [3]. In addition, these systems aim to control the product selectivity and the rate of catalytic reactions by noncovalent interactions, mimicking the natural enzymatic catalysis. Especially macrocycles provide a convenient method for the construction of supramolecular catalytic systems since macrocycles can act as both a stabilizer and electron transporter in supramolecular systems. In addition to that, the main advantages of the macrocycles in photocatalysis including: i) substrate selectivity, ii) controllability of the rate of photochemical reactions, iii) the close proximity to substrates in a confined space, iv) the generation of a stabilized high-energy transition state, and v) macrocyclic-cavity-size-dependent selective arrangement of one or two substrates within the cavity for photocatalysis. Therefore, this review will focus on: i) the role of the supramolecular system in mediating the photocatalytic selectivity, yield, and the rate of the photocatalytic products and ii) macrocycle-assisted hybrid materials that have been exploited for photocatalytic applications, including photocatalytic dye degradations and hydrogen evolution. To successfully perform supramolecular photocatalytic reactions, various photophysical and photochemical properties of the host–guest system need to be considered [4]: i) the structural rigidity caused by supramolecular host–guest complexation, ii) that a restricted space, size, and volume could lead to three-dimensional translation (rotation, vibration, and translation) of the guests within the host cavities, and iii) the dynamic properties of the guest within the supramolecular system. Based on the above-mentioned approaches, organic and inorganic template-assisted diverse supramolecular assemblies have been constructed to optimize the photochemical reactivity as well as regio- and enantioselectivity [5–6]. In addition, to understand the excited-state properties of the guest via photophysical analyses is relatively important. Hence, this review aims to summarize the photochemical transformation and catalytic reactivity of different guests within the various macrocyclic hosts of various sizes and shapes based on molecular recognition as well as the role of macrocyclic-host-assisted hybrid materials in energy transfer. To date, several supramolecular hosts have been developed that can provide defined properties of, and exert catalytic control on reactive substrates (guests) [7–9]. To keep the clarity, we categorized this into the five most universally used important supramolecular hosts (Figure 1); crown ethers (CEs), cyclodextrins (CDs), cucurbiturils (CBs), calixarenes (CAs), and pillararenes (PAs) for various photocatalytic reactions. However, biomolecules and other supramolecular-host-related photocatalytic reactions are not included in this minireview.

Generally, a photocatalytic reaction with a rate acceleration facilitated by supramolecular host–guest interactions and hybrid materials are typically assisted by noncovalent forces, including Coulomb and van der Waals forces, hydrogen bonding, and π–π interactions. For potential supramolecular photocatalysts, they should satisfy the following criteria: i) a reaction-ready configuration, i.e., a substrate orientation in a configuration favorable for photochemical transformations, ii) in the supramolecular photocatalytic reaction (within the cavity), the quantum yield should be higher than both the unimolecular and bimolecular photoreaction quantum yield in an isotropic medium, and iii) the substrate and the products should be released from the supramolecular system via a dynamic exchange, which directs to a better yield and catalytic efficiency. To strengthen the above concepts, supramolecular photocatalysis needs a comprehensive investigation of the kinetic and thermodynamic aspects relating to the host–guest interactions and photochemical transformation efficiency of the substrate [10–11]. Thus, this review highlights the supramolecular photocatalytic substrate transformation involving various stoichiometric host–guest complexes (1:1, 2:1, 1:2, and 2:2). For the 1:1, 2:1, and 1:2 host–guest complexes, the thermodynamic association (*K*_a_) and dissociation constants (*K*_d_) for the reactive guests and photoproducts are crucial for the fundamental understanding of supramolecular noncovalent interactions [12–13]. In addition, during the catalytic process, the reactive guest should have a greater binding affinity or the photoproducts should have the lowest binding affinity with the supramolecular host to avoid product inhibition, so as to produce a better catalytic turnover efficiency [14–15]. However, it is quite complicated to determine the kinetic rates of the forward and reverse reactions. Instead, the supramolecular photochemical transformation efficiency is calculated via quantum yield and reaction velocity experiments. In addition, the thermodynamic and kinetic functions of the supramolecular systems can be authenticated by isothermal calorimetric titration or fluorescence titration and stopped-flow analysis. Furthermore, the quantum yield and reaction velocity of the photocatalysis can be verified by actinometry and UV–vis measurements. Therefore, with the aid of various analytical and spectroscopic studies, we can easily gain information on host–guest interactions in supramolecular systems as well as the photocatalytic reaction efficiency.

## Review

### Macrocycle-promoted photocatalysis

#### Photocatalysis based on crown ethers and crown ether derivatives

CEs are a class of macrocycles that consist of a ring with a repeating unit. The term "crown" is derived from the resemblance of the crown ether when bound to a cation with a crown sitting on a head. The most common crown ethers are those of ethylene oxide. The exterior of the ring is hydrophobic, while the oxygen atoms within the ring can strongly coordinate to a cation, forming complexes. Crown ethers also contain a series of derivatives, such as aza-crowns, where the ether oxygen atoms are replaced by amino groups. A well-known tetrazacrown is cyclen. Due to the excellent host–guest properties of crown ethers, they play a very important role in the construction of functional photocatalytic systems [16–17]. The diversity of crown ether-based photocatalytic reactions is relatively low, involving mainly photocycloaddition reactions.

**Figure 2:**
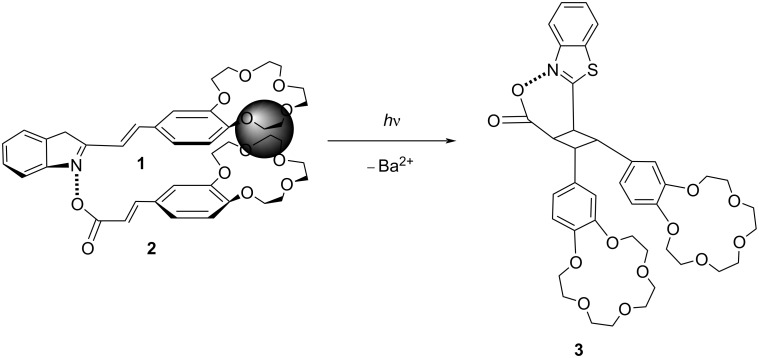
Ba^2+^-induced intermolecular [2 + 2]-photocycloaddition of crown ether-functionalized substrates **1** and **2** to form cycloadduct **3**. Republished with permission of The Royal Society of Chemistry from [18] (“Supramolecular photochemical synthesis of an unsymmetrical cyclobutane” by O. Fedorova et al., Photochem. Photobiol. Sci. vol. 6, issue 10, © 2007); permission conveyed through Copyright Clearing Center, Inc.

Saltiel and co-workers reported the regiospecific intermolecular [2 + 2]-photocycloaddition based on a supramolecular assembly of two different crown ether-substituted molecules, styrylbenzothiazole and cinnamic acid [18]. In this system, the Ba^2+^ cation can preorganize the two crown ether-substituted molecules into a supramolecular complex (Figure 2), which was further stabilized by hydrogen bonding and π–π interactions between them. These nonbonding interactions could allow the double bonds to be placed at an optimal distance for the efficient [2 + 2]-photoadduct. However, this adduct could not be observed in the absence of the metal cation, indicating the importance of the preorganization of the substrates with Ba^2+^ and crown ether, leading to the formation of cyclobutane in a high yield.

**Figure 3:**
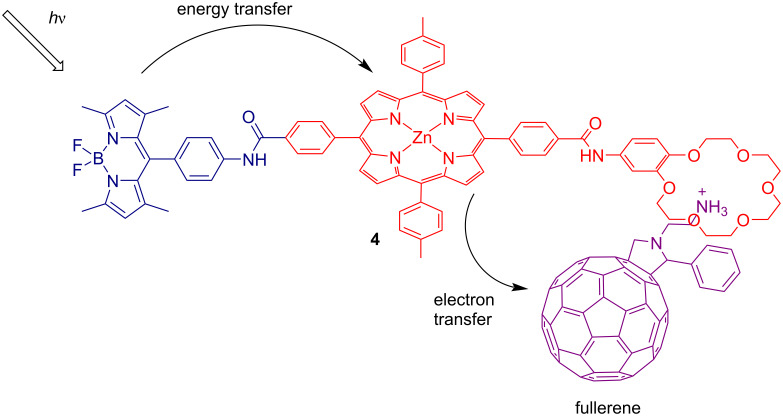
Energy transfer system constructed of a BODIPY–zinc porphyrin–crown ether triad assembly bound to a fulleropyrrolidine. Adapted with permission from [19], Copyright 2009 American Chemical Society.

D’Souza et al. constructed a photosynthetic triad **4** to mimic the photosynthetic reaction center [19]. A BODIPY, an ammonium-functionalized fullerene, and a zinc porphyrin functioned as the energy donor, electron acceptor, and electron donor, respectively (Figure 3). A benzo-18-crown-6 derivative was connected to the porphyrin ring, which could form a supramolecular complex with fullerene through dipole interactions. The formed supramolecular self-assembly resulted in a high energy-transmission efficiency (over 97%) between the BODIPY and the zinc porphyrin. The excited zinc porphyrin further transferred the electron to the fullerene, yielding a charge-separated state, with a long lifetime of 23 µs, indicating the charge stabilization of the supramolecular assembly. Thus, an artificial photocatalytic system that can mimic the photosynthetic reaction center for photosynthesis could be realized.

**Figure 4:**
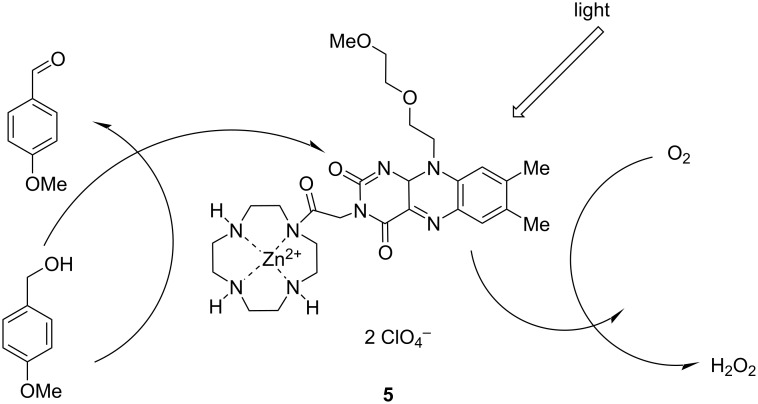
The sensitizer **5** was prepared by a flavin–zinc(II)–cyclen complex for the photooxidation of benzyl alcohol. Figure 4 reproduced from [20]. Copyright © 2004 WILEY‐VCH Verlag GmbH & Co. KGaA, Weinheim. Used with permission from R. Cibulka et al., Catalytic Photooxidation of 4‐Methoxybenzyl Alcohol with a Flavin–Zinc(II)‐Cyclen Complex, Chemistry – A European Journal, John Wiley and Sons.

König and co-workers demonstrated the use of a flavin–Zn(II)–cyclen assembly (Figure 4) to convert benzyl alcohol to benzaldehyde upon irradiation [20]. This reaction could be performed in both organic and aqueous phases, with a photooxidation quantum yield of 3.8 × 10^−2^ and 0.4, respectively. In contrast, for flavins without the zinc(II)–cyclen unit, only small amounts of product were observed, and the quantum yield was 30 times lower compared to that of the assembly with the flavin chromophore possessing a binding site. The mechanism may be explained as follows: the zinc(II)–cyclen complex can absorb light and facilitate the intramolecular electron transfer from benzyl alcohol to the excited flavin, and thus the benzaldehyde and the photoreduced flavin were produced. The study indicates the significance of connecting a photosensitizer and a substrate with suitable binding sites for photochemical transformations.

#### Cyclodextrin-based photocatalysis

CDs represent a family of macrocyclic compounds in which the ᴅ-glucopyranose units are connected to each other via an α-1,4-glycosidic bond. The inside of a cyclodextrin is a hydrophobic cavity, and the outside possesses the polyhydroxy structure. This unique asymmetric barrel-shaped cavity allows them to combine with various guests. The driving forces for the formation of cyclodextrin-based supramolecular complexes are hydrophobic and van der Waals interactions between the cavity and the hydrophobic part of the guests. α-, β-, and γ-CDs are the most common macrocycles, consisting of 6, 7, and 8 ᴅ-glucopyranose residues, respectively. Due to the differences in the cavity size, each cyclodextrin has the ability to form a complex with a specific guest, and the binding force depends on the size of the host and guest as well as the degree of geometric complementarity [21–22]. CDs can recognize guests in both aqueous and solid environments, so that they can be used as an excellent host for the construction of photocatalytic systems. CD-based host–guest systems can realize various types of photochemical reactions, including photoisomerization, photocyclodimerization, and H_2_ evolution [7,23].

**Figure 5:**
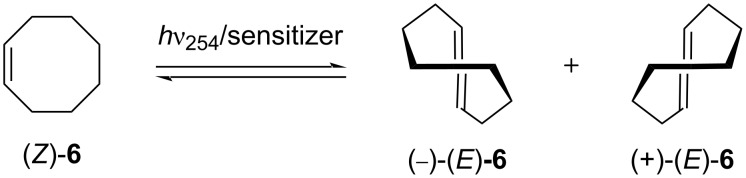
Enantiodifferentiating *Z*–*E* photoisomerization of cyclooctene sensitized by a chiral sensitizer as the host. Adapted with permission from [24], Copyright 2000 American Chemical Society.

**Figure 6:**
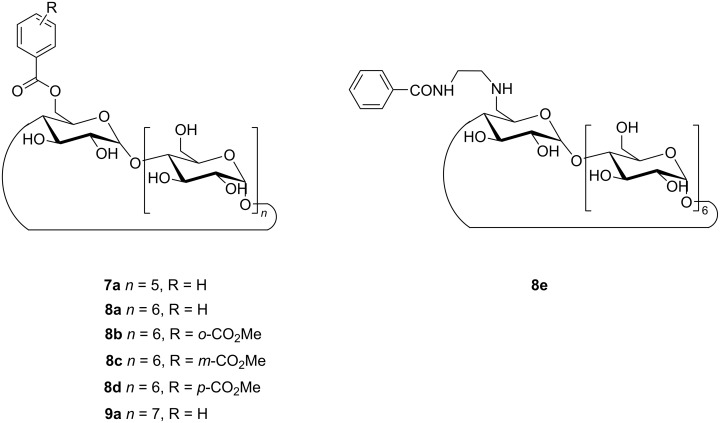
Structures of the modified CDs as chiral sensitizing hosts. Adapted with permission from [24], Copyright 2000 American Chemical Society.

Chirality induction in a prochiral guest via photochemical reactions is a delightful approach. This can not only transfer the chirality of the host cavity to the molecular photoproduct via excited-state supramolecular chiral interactions but can also improve the photoenantiodifferentiating proficiency of the host. To prove this concept, Inoue et al. investigated the photoisomerization of (*Z*)-cyclooctene ((*Z*)-**6**) to the chiral *E*-isomer (*E*)-**6** in an aqueous methanol solution through host–guest interactions between the modified CDs **7a**–**9a** as sensitizing hosts and (*Z*)-**6** as the guest (Figure 5 and Figure 6) [24]. The compounds **8a**, **8b**, and **8e** could yield a higher enantiomeric excess (10.7% ee in 2 min at 25 °C, host occupancy 91%, 23.9% ee in 25 min at 40 °C, host occupancy 98%, and 8.1% ee in 30 min at 25 °C, host occupancy 95%) in MeOH/H_2_O solutions (0.25:0.75, 0.5:0.5, and 0:1, respectively). This may be due to fact that the CD-functionalized sensitizing group could increase the product yield through excited-state supramolecular interactions within the cavity. In addition, the spacer attached to the primary hydroxy rim of the CDs could also be optimized, leading to the conformational fixation of both the sensitizer and the guest within the chiral cavity and producing the high ee values. For **8c** and **8d**, the self-inclusion of the aromatic chromophore might be interrupted, resulting in the long distance from the guest and a less efficient asymmetric induction. In contrast, α-**7a** and γ-CD **9a** produced (*E*)-**6** with a poor ee (<3 and 5%, respectively) because of the size mismatch between (*Z*)-**6** and this host cavity. These results suggested that the ee value of the supramolecular photochirogenesis system depend on various main factors, such as the percentage of the host occupancy, the temperature, and the solvent but not on the entropy of the system.

**Figure 7:**
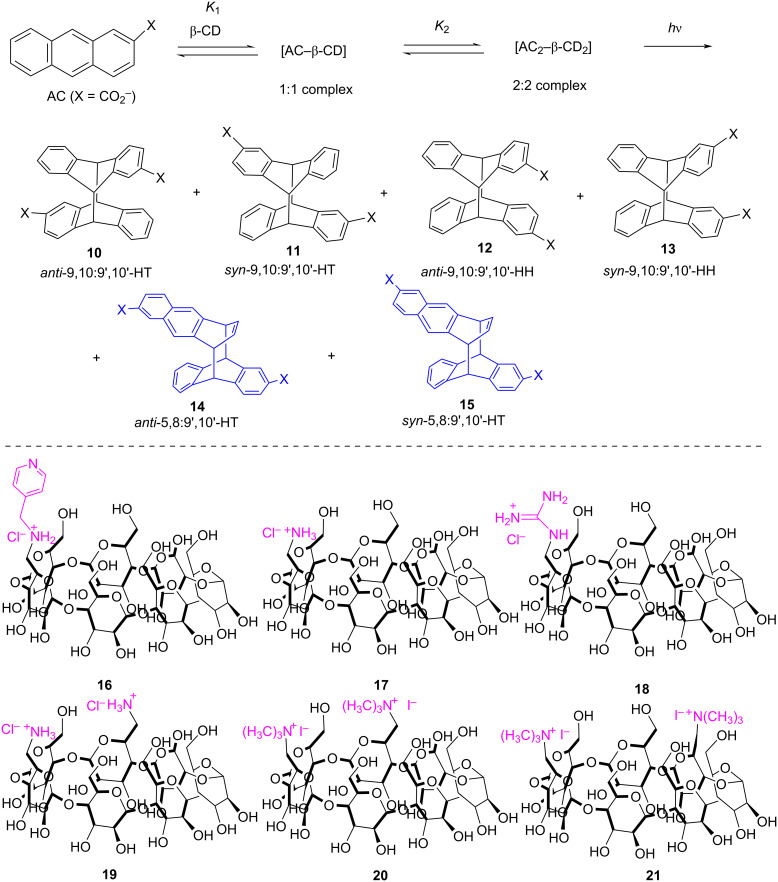
Supramolecular 1:1 and 2:2 complexations of AC with the cationic β-CD derivatives **16**–**21** and subsequent photocyclodimerization to give the classical 9,10:9',10'-cyclodimers **10**–**13** and the nonclassical 5,8:9',10'-cyclodimers **14** and **15**. Adapted with permission from [25], Copyright 2018 American Chemical Society.

In continuation of their previous work, Inoue, Yang, et al. reported an alternative approach so that the chiral slipped 5,8:9',10'-cyclodimers **14** and **15** were selectively obtained over the conventional 9,10:9',10'-cyclodimers by the supramolecular photocyclodimerization of 2-anthracenecarboxylate (AC) with the aid of β-CD-functionalized cationic derivatives as chiral catalysts (Figure 7) [25]. Due to the electrostatic interactions between cationic CD and anionic AC, the 1:1 (photoinert) and 2:2 (photoreactive) complexes (*K*_1_ = 33100 M^−1^ and *K*_2_ = 1480 M^−1^, respectively) could be formed, which have a stronger binding affinity than the native CD (*K*_1_ = 3800 M^−1^ and *K*_2_ = 150 M^−1^). Such interactions considerably enhance the population of the 2:2 complex (partially stacked in a slipped *anti*- or *syn*-head-to-tail (HT) fashion) to afford the successive photocyclodimerization, which mimics a catalytic antibody. Therefore, various head-to-head (HH)- or HT-photodimers could be obtained. Particularly, the **21**–AC complex could be involved in the photolysis and produced the slipped chiral cyclodimers **14** (80–85% yield, 52–71% ee) and **15** (15–20% yield, 7–17% ee), respectively, with a perfect selectivity, compared to that of the other hosts, at every temperature tested. Meanwhile, the conventional cyclodimers **10**–**13** were noticeably suppressed. By tuning the various internal and external factors of this photocyclodimerization reaction (such as the temperature, pressure, salt concentration, and host substituents), the regio- and enantioselectivity could considerably be varied to 71% ee for **14** and 45% ee for **15**, respectively. In this supramolecular system, the 2:2 complex could instantly be disassembled by a volume-expanding photocyclodimerization and recycled for repeated use via the dimerization of 1:1 complexes.

In 2018, Wu, Yang, et al. also reported the photocyclodimerization of AC with the aid of 6^A^,6^X^-diguanidio-γ-CDs [26]. The γ-CDs and AC could form stable 1:2 ternary complexes. Moreover, they also demonstrated the entropic contribution during the controllable photocyclodimerization of AC.

More recently, Inoue, Yang, et al. reported that the AC could be obtained regio- and enantioselectively by the photocyclodimerization, with the aid of β-CD dimers with a defined stereochemistry as catalysts [27]. The stereochemical properties were sensitive to the linker size and length, and the yield of the *syn*-head-to-tail-9,10:9',10'-cyclodimer could reach 97–98%.

**Figure 8:**
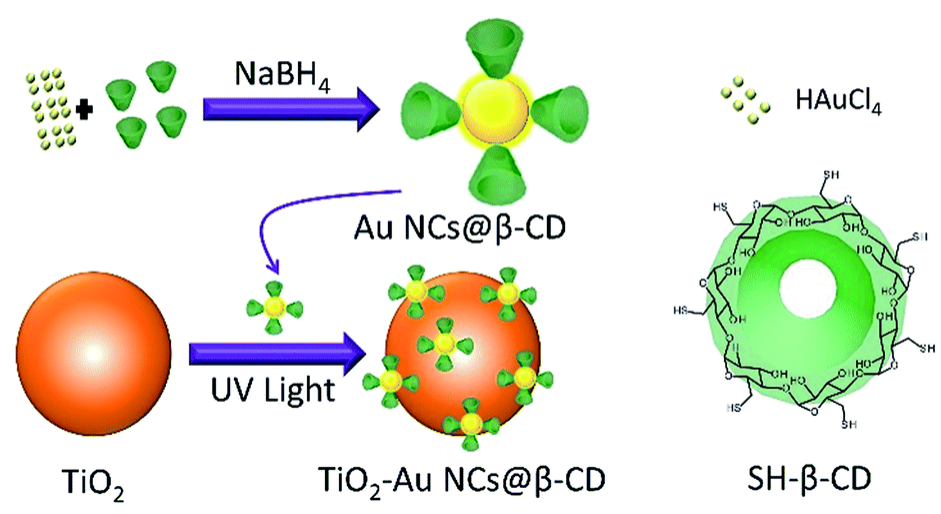
Construction of the TiO_2_–AuNCs@β-CD photocatalyst. Republished with permission of The Royal Society of Chemistry from [28] (“Cyclodextrin–gold nanocluster decorated TiO_2_ enhances photocatalytic decomposition of organic pollutants” by H. Zhu et al., J. Mater. Chem. A vol. 6, © 2017); permission conveyed through Copyright Clearing Center, Inc.

Traditional thiol-functionalized organic ligands decorated on the surface of gold nanoclusters (AuNCs) tend to generate a physical barrier in photocatalytic reactions. To address this issue, Chen and co-workers prepared a TiO_2_–AuNCs@β-CD-based hybrid material on TiO_2_ and β-CD-protected AuNCs, which can potentially be used for the photocatalytic degradation of methyl orange (MO) dye (Figure 8) [28]. Upon UV-light irradiation, this hybrid material rapidly degraded MO (≈98%) within 10 min, whereas freshly prepared TiO_2_ reached a value of ≈47%. This may be due to the synergic effect of β-CD and the AuNCs, which relies on two aspects: i) the host–guest interaction between β-CD and MO could lead to the Au cores being directly accessible for MO and ii) the Au cores could perform as electron acceptors (Au^+^ to Au^0^), which prevents the recombination of the electron–hole pairs produced by TiO_2_. Such a system generated more excited electrons for the generation of reactive oxygen species (ROS) and also acted as an effective reaction center for dye degradation. Interestingly, the photodegradation rate was significantly improved (≈95%) by adding ethylenediaminetetraacetic acid (EDTA, 0.02 mM) as the hole scavenger. To evaluate the stability of the TiO_2_–AuNCs@β-CD catalyst, recycling experiments were conducted. These results implied that the catalyst efficiency is almost constant even after five cycles, but the rate of the reaction considerably decreased from the first cycle (0.31 min^−1^) to the fifth cycle (0.15 min^−1^).

**Figure 9:**
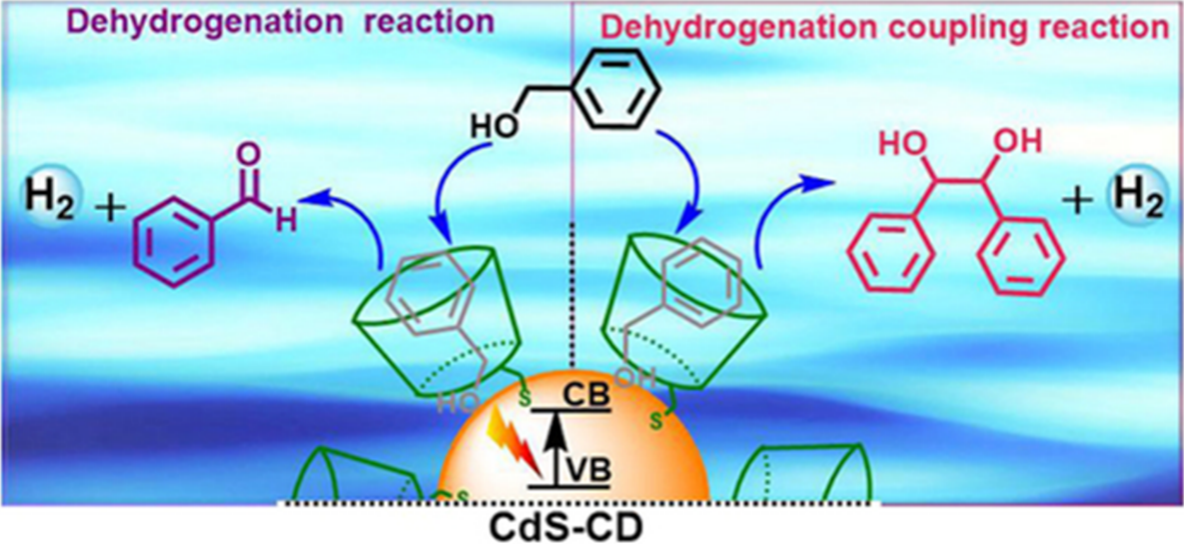
Visible-light-driven conversion of benzyl alcohol to H_2_ and a vicinal diol or to H_2_ and benzaldehyde by CdS–CD. This figure has been published in CCS Chemistry [2020]; [β-Cyclodextrin Decorated CdS Nanocrystals Boosting the Photocatalytic Conversion of Alcohols] is available online at [DOI; https://www.chinesechemsoc.org/doi/10.31635/ccschem.020.201900093].

Zhang, Lu, et al. prepared β-CD-decorated CdS nanocrystals (CdS–CD, Figure 9) via a ligand-exchange reaction between thiol-functionalized β-CD and oleic acid-protected CdS nanocrystals [29]. These spherical CdS–CD nanoparticles could be employed as a photocatalyst for the dehydrogenation of alcohols to aldehydes (at a low concentration of the reactant of 1 mM, ≥92% selectivity) or diols (at a high concentration of the reactant of 300 mM, ≥93% selectivity), with H_2_ liberation being achieved by visible-light irradiation in an aqueous solution. In comparison, CdS–CD was a highly efficient photocatalyst for benzyl alcohol dehydrogenation (77 µmol H_2_ in 180 h) compared to the CD-free CdS (5.4 µmol H_2_ in 30 h, saturated), which might be due to the selective host–guest interactions between β-CD and the reactants. In addition, the captured reactants were in close proximity to the CdS nanocrystal surface. Consequently, the barrier of the surface ligands on the photocarrier transport could be reduced.

**Figure 10:**
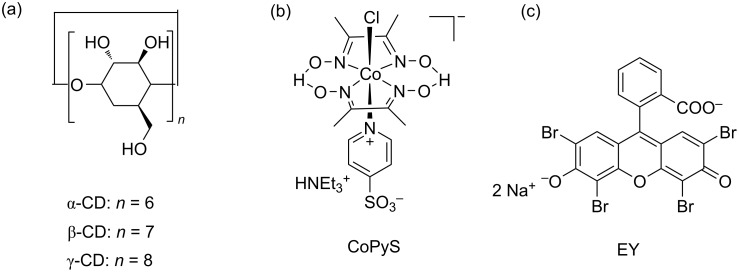
(a) Structures of CDs, (b) CoPyS, and (c) EY. Republished with permission of The Royal Society of Chemistry from [30] (“Host–guest chemistry between cyclodextrin and a hydrogen evolution catalyst cobaloxime” by M. Kato et al., New J. Chem. vol. 43, © 2019); permission conveyed through Copyright Clearing Center, Inc.

Recently, Kato, Yagi, et al. reported that the visible-light-driven hydrogen evolution of the supramolecular system could be achieved by the host–guest complexation between the cobaloxime CoPyS and γ-CD (Figure 10) [30]. Upon visible-light irradiation, the CoPyS:γ-CD 1:1 complex exhibited an enhanced photocatalytic H_2_ evolution ability in the presence of eosin Y (EY) dye (9.9 ± 0.2 mmol H_2_ in 60 min) in a neutral aqueous solution compared to that of a mixed α-,β-CD:CoPyS complex and free CoPyS (8.5 ± 0.3 mmol H_2_ in 60 min). This significant H_2_ evolution might be due to the fact that the electrostatic repulsion between negatively charged CoPyS and EY was suppressed by the inclusion of γ-CD.

#### Calixarene-based photocatalysis

CAs are derived from the shape of the chalice. They can be obtained by bridging phenolic units through methylene groups in the *meta*-position. Their rigid goblet structure has two edges. The wider upper edge is hydrophobic, while the narrower lower edge is hydrophilic due to the presence of phenolic oxygen atoms. Depending on the number of phenol units, calixarenes have a variable cavity size, and thus are able to combine with different guests. Therefore, calixarenes can be used as effective supramolecular hosts to recognize guests through hydrophobicity, π–π stacking, cation–π interactions, ion–dipole interactions, etc. [31–32]. Due to the cavity-shape limitation of calixarenes, most calixarene-based photocatalytic systems are mainly based on the fabrication of hybrid materials for energy transfer and photocatalysis [33–35].

**Figure 11:**
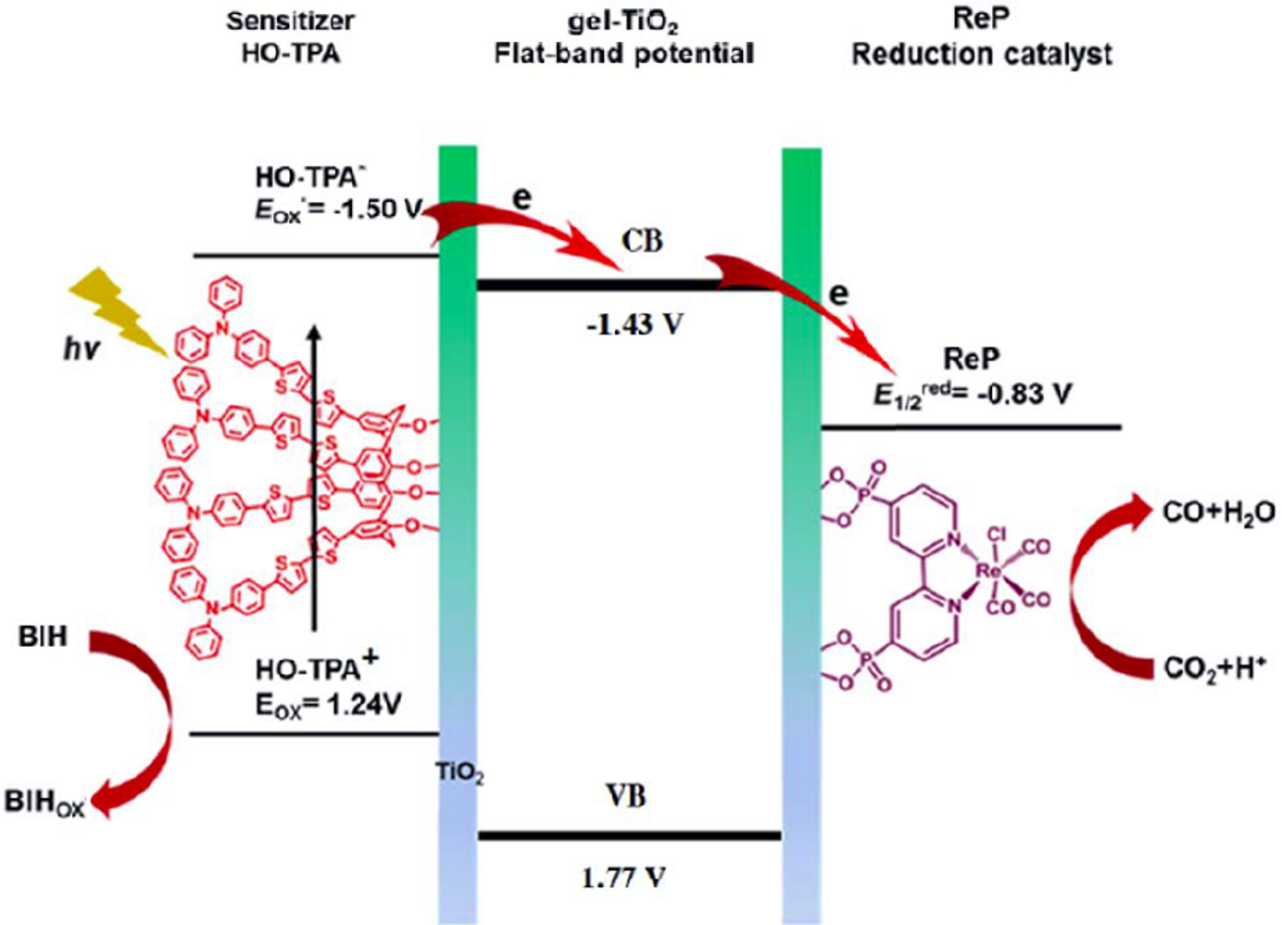
Conversion of CO_2_ to CO by ReP/HO-TPA–TiO_2_. Republished with permission of The Royal Society of Chemistry from [36] (“A porous hybrid material based on calixarene dye and TiO_2_ demonstrating high and stable photocatalytic performance” by Y. Chen et al., J. Mater. Chem. A vol. 7, © 2019); permission conveyed through Copyright Clearing Center, Inc.

Su and co-workers reported a hybrid material based on a calixarene-modified dye and TiO_2_ (HO-TPA–TiO_2_) [36]. The calixarene could combine with TiO_2_, providing efficient electron transfer between them (Figure 11). The TPA–TiO_2_ system exhibits an efficient H_2_ evolution activity (618.3 mmol⋅g^−1^⋅h^−1^) and an excellent stability (with a turnover number (TON) of 6417 after 75 h). Moreover, when the electron donor 1,3-dimethyl-2-phenyl-1,3-dihydrobenzimidazole (BIH) was added, the hybrid material functioned as an efficient photocatalyst for the reduction of CO_2_ to CO with 0.26 wt % ReP as a cocatalysts. The TON was 534 (2.19 mmol⋅g^−1^⋅h^−1^), without a significant levelling-off tendency for 26 h. This work provided a novel method for developing organic–inorganic hybrid materials based on macrocycles for efficient photocatalysis.

**Figure 12:**
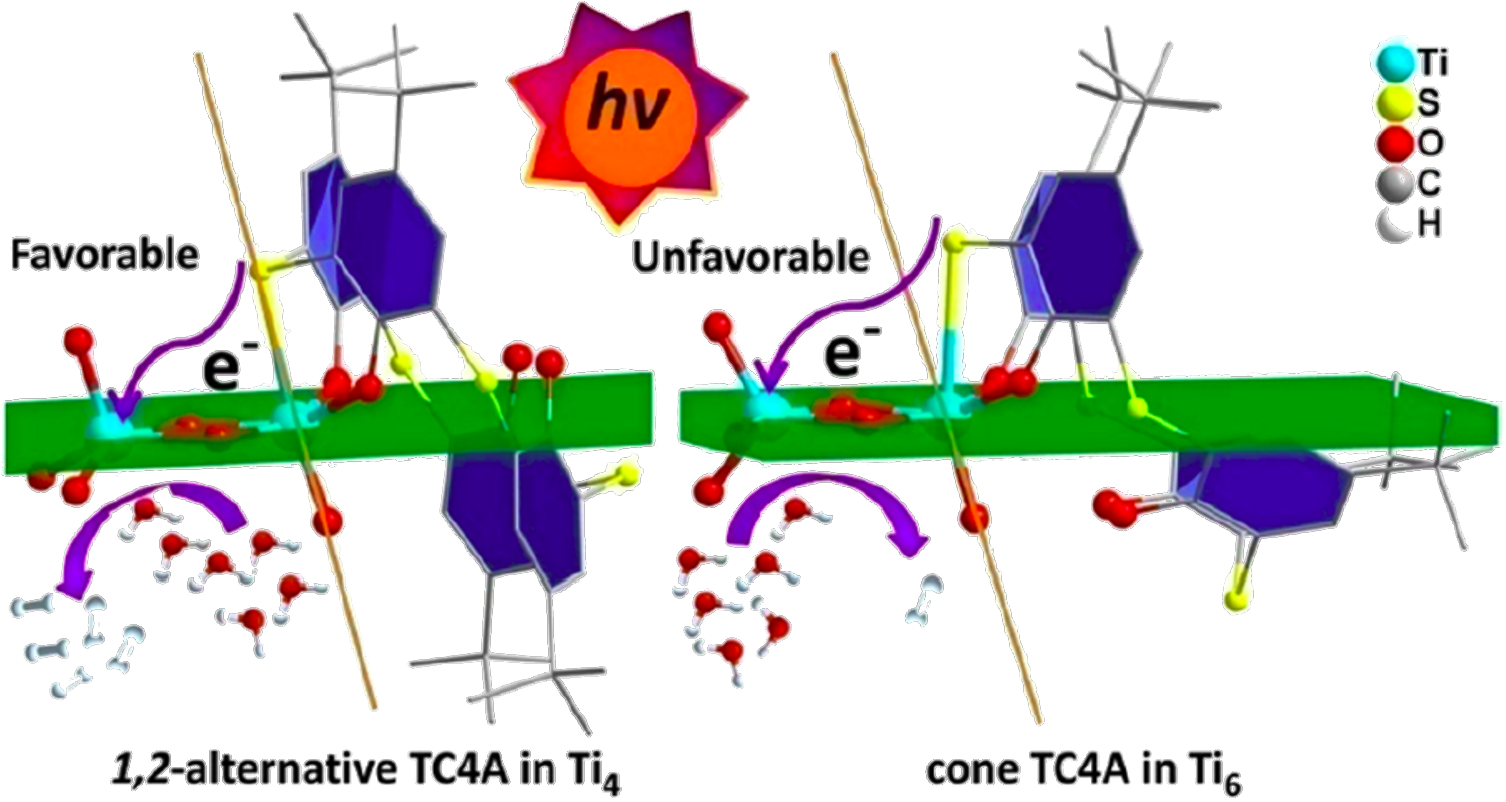
Thiacalix[4]arene-protected TiO_2_ clusters for H_2_ evolution. Reprinted with permission from [37], Copyright 2020 American Chemical Society.

In a similar work, Zheng and co-workers reported two types of thiacalix[4]arene-protected titanium–oxo clusters [37]. In that work, *p*-*tert*-butylthiacalix[4]arene (H_4_TC4A) was introduced to tetranuclear and hexanuclear clusters Ti_4_ and Ti_6_, respectively (Figure 12). The formed hybrid materials had band gaps of 2.19 eV (Ti_4_) and 2.24 eV (Ti_6_). The different Ti–S coordination modes between H_4_TC4A–Ti_4_ and H_4_TC4A–Ti_6_ result in different photocatalytic activities. The H_2_ evolution of H_4_TC4A–Ti_4_ was almost 5 times that of H_4_TC4A–Ti_6_ (73.56 μmol⋅g^−1^⋅h^−1^ vs 15.16 μmol⋅g^−1^⋅h^−1^). This work emphasized the importance of structure–efficiency relationships of titanium–thiacalix[4]arene-based clusters for H_2_ evolution, and it also provides a useful method for tuning the band gaps of the catalysts.

**Figure 13:**
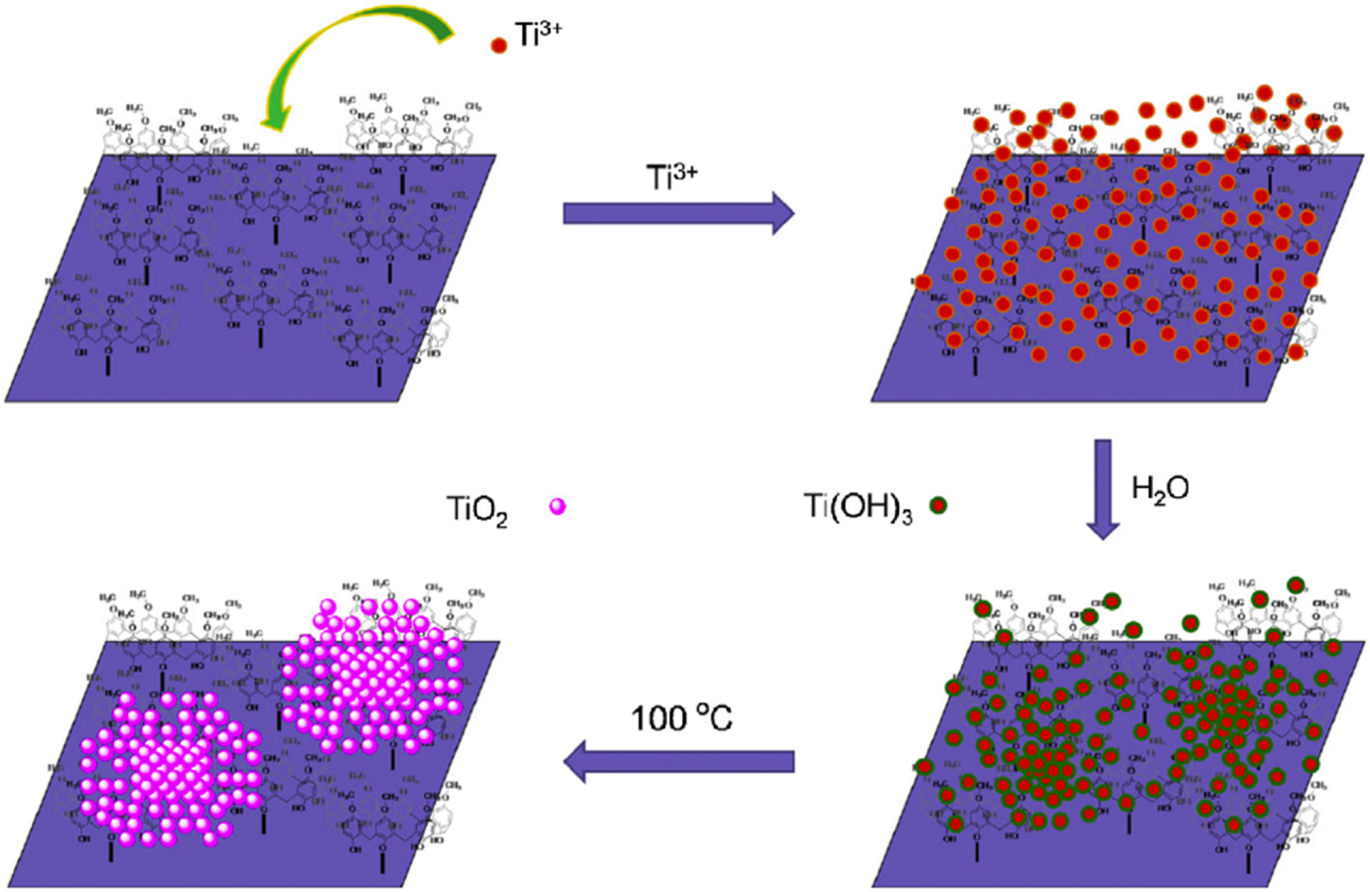
4-Methoxycalix[7]arene film-based TiO_2_ photocatalytic system. Reprinted from [38], Materials Today Chemistry, vol. 1–2, by R. Zhou, M. P. Srinivasan “Fabrication of anti-poisoning core-shell TiO_2_ photocatalytic system through a 4-methoxycalix[7]arene film’’ pages 1–6, Copyright (2016), with permission from Elsevier.

Apart from reducing the distance between the electron donor and acceptor, calixarenes can also promote the charge separation to enhance the electron transfer [38]. Zhou and co-workers employed 4-methoxycalix[7]arene and quartz beads as the host and packing material, respectively (Figure 13). The formed packed bed could then immobilize TiO_2_. Therein, the 4-methoxycalix[7]arene not only led to a decrease in the band gap of TiO_2_ but also prohibited the recombination of electron–hole pairs. Consequently, a high activity of TiO_2_ could be achieved, and the decolorization of methyl orange was realized efficiently.

#### Cucurbituril-based photocatalysis

CBs are a type of macrocyclic molecules made of glycoluril linked by methylene bridges. The name is derived from the resemblance of the pumpkin of the Cucurbitaceae family. The oxygen atoms are located along the edges of the band, which can identify positively charged guests through ion–dipole and hydrogen bond interactions. The internal cavity exhibits a strong hydrophobicity. Depending on the size and shape of the guest molecules, binary 1:1 or ternary 1:1:1 complexes can be formed. In view of the unique host–guest binding characteristics, cucurbituril is also often used to construct photocatalytic systems [39–41]. The large and hydrophobic cavities of the CBs provide a suitable environment for various photochemical reactions, including photopolymerization, photohydrolysis, and photodimerization [42–46].

**Figure 14:**
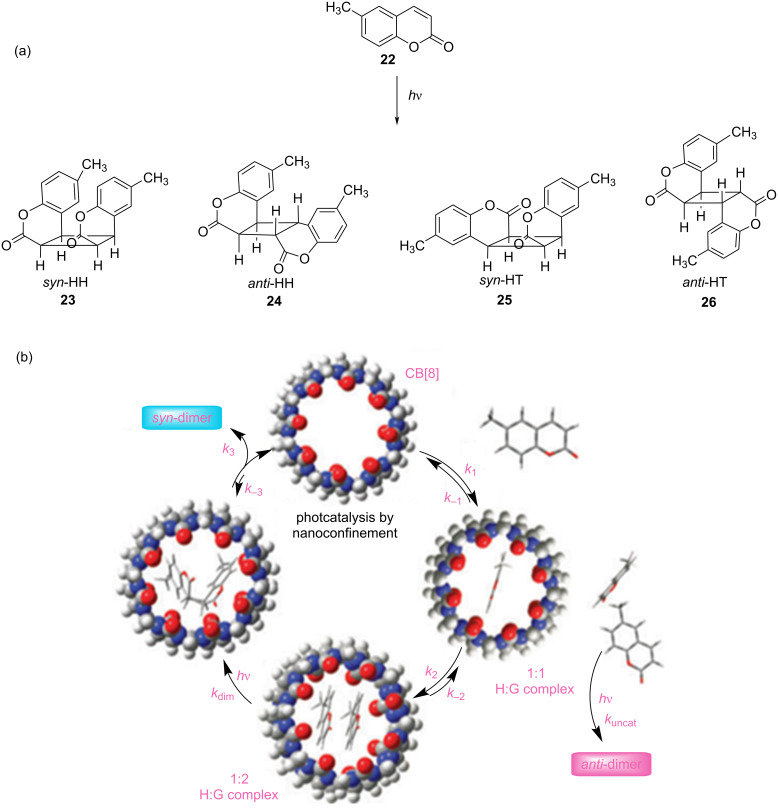
(a) Photodimerization of 6-methylcoumarin (**22**). (b) Catalytic cycle for the photodimerization of **22**, mediated by CB[8]. Republished with permission of The Royal Society of Chemistry from [47] (“Supramolecular photocatalysis by confinement—photodimerization of coumarins within cucurbit[8]urils” by B. C. Pemberton et al., Chem. Commun. vol. 46, © 2010); permission conveyed through Copyright Clearing Center, Inc.

Chemical transformations assisted by nanosized reaction vessels have gained attention in the field of supramolecular photocatalysis. Therefore, Sivaguru et al. performed the photodimerization of coumarin **22** with a catalytic amount of CB[8] as nanosized reaction vessels, which produced *syn*-photodimers **23** and **25** under light-irradiation as well as in sunlight (Figure 14) [47]. By gradually increasing the molar ratio of CB[8] (from 10 to 100 mol %), the conversion yield of the *syn*-products **23** (*syn*-HH) and **25** (*syn*-HT) in an aqueous solution increased from 23 to 76% (60 min, under N_2_, O_2_, and air), with a high selectivity (>99%). However, for reactions without adding CB[8] as a catalyst, the conversion yield was below 10%. In addition, the fluorescence enhancement was also observed at a host/guest CB/**22** ratio of 1:2, which revealed that the singlet spin-state of coumarin could be involved in the photodimerization process within the CB[8] cavity and that the triplet state could be shielded from external quenchers (O_2_ as a triplet quencher). These results also indicated that photodimerization could occur within the CB[8] cavity via host–guest interactions, and thus predominantly produced the *syn*- instead of the *anti*-dimers.

**Figure 15:**
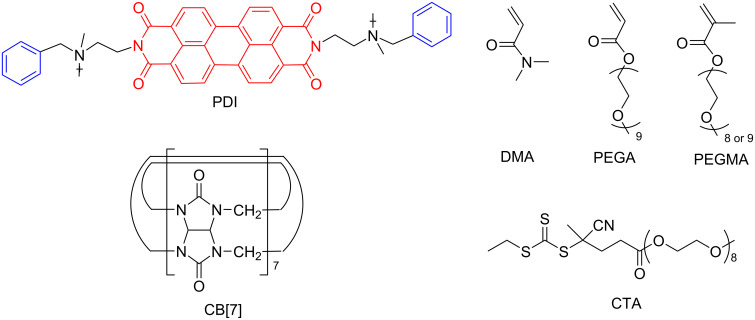
Formation of a supramolecular PDI–CB[7] complex and structures of monomers and the chain transfer agent. Republished with permission of The Royal Society of Chemistry from [48] (“Visible light induced aqueous RAFT polymerization using a supramolecular perylene diimide/cucurbit[7]uril complex” by Y. Yang et al., Polym. Chem. vol. 10, © 2019); permission conveyed through Copyright Clearing Center, Inc.

An alternative approach introduced by An et al. is about the 2:1 CB[7]–perylene diimide (PDI) host–guest-assisted reversible addition–fragmentation chain transfer (RAFT) photopolymerization under visible-light irradiation in an aqueous solution [48]. Briefly, the supramolecular PDI–CB[7] complex in Figure 15 acts as a photocatalyst at a low concentration (1 ppm relative to the monomer), which could be utilized for the preparation of a series of homo- or block copolymers with an ultrahigh molecular weight (≈10^6^ g⋅mol^−1^) and a reasonably low dispersity (*Đ* ≥ 1.33) by using various monomers. Triethanolamine (TEOA) was used as an electron donor to initiate the photoinduced electron transfer to the PDI–CB[7] system, and thus to start the polymerization reaction. In addition, the rate of this supramolecularly driven photopolymerization (0.0305 min^−1^) could be enhanced by decreasing the aggregation of PDI via host–guest interactions.

**Figure 16:**
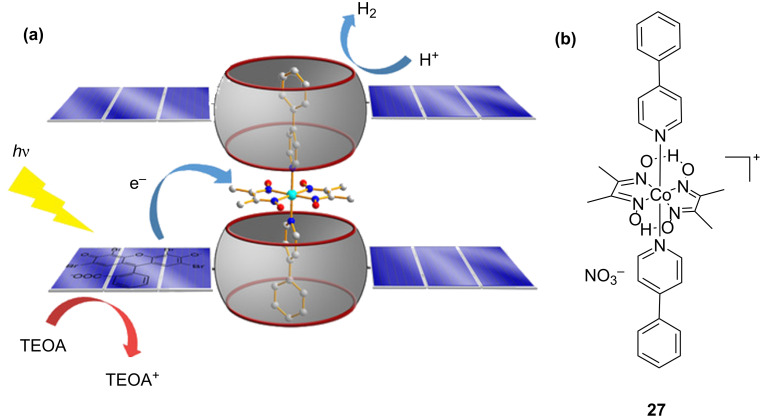
Ternary self-assembled system for photocatalytic H_2_ evolution (a) and structure of **27** (b). Figure 16 reproduced from [49]. © 2019 Wiley‐VCH Verlag GmbH & Co. KGaA, Weinheim. Used with permission from D. Song et al., Orthogonal Supramolecular Assembly Triggered by Inclusion and Exclusion Interactions with Cucurbit[7]uril for Photocatalytic H_2_ Evolution, ChemSusChem, John Wiley and Sons.

Recently, Wang and co-workers fabricated the orthogonal supramolecular ternary self-assembled system **27**@CB[7]/EY via host–guest interactions between CB[7], the [Co(dmgH)_2_(4-ppy)_2_]NO_3_ (**27**, dmgH_2_ = dimethylglyoxime, 4-ppy = 4-phenylpyridine) guest, and the EY photosensitizer, respectively (Figure 16) [49]. When mixed with the sacrificial electron donor TEOA in an anaerobic H_2_O/CH_3_CN 1:1 solution, this supramolecular system **27**@CB[7]/EY could realize an efficient H_2_ evolution under irradiation with visible light. The TON of the system is 51, and the turnover frequency (TOF) is 43 h^−1^, which is a 6-fold and a 3-fold, respectively, of that without CB[7]. The enhanced H_2_ evolution efficiency may be due to the linking effect of CB[7] with EY and **27** and due to their spatial proximity.

#### Pillararene-based photocatalysis

Pillararenes are a class of pillar-shaped macrocyclic hosts with the methylene group bridged at the *para*-position of 2,5-dialkoxybenzene. The size of the cavity can be adjusted by the number of repeating units, i.e., pillar[5]arene or pillar[6]arene. Pillararenes have electron-rich cavities that facilitate the combination with various electron-deficient guest molecules, such as alkylammonium, pyridinium, and imidazolium cations. Compared to other macrocycles, pillararenes exhibit a high degree of symmetry and rigidity, which gives them a better binding ability to guest molecules. They can also be easily functionalized, providing a variety of modified functional derivatives [50–52]. Therefore, these superior characteristics give pillararenes certain advantages for constructing photocatalytic systems. The photocatalytic system based on pillararene is a relatively new field, and only a few examples have been reported [53–54].

**Figure 17:**
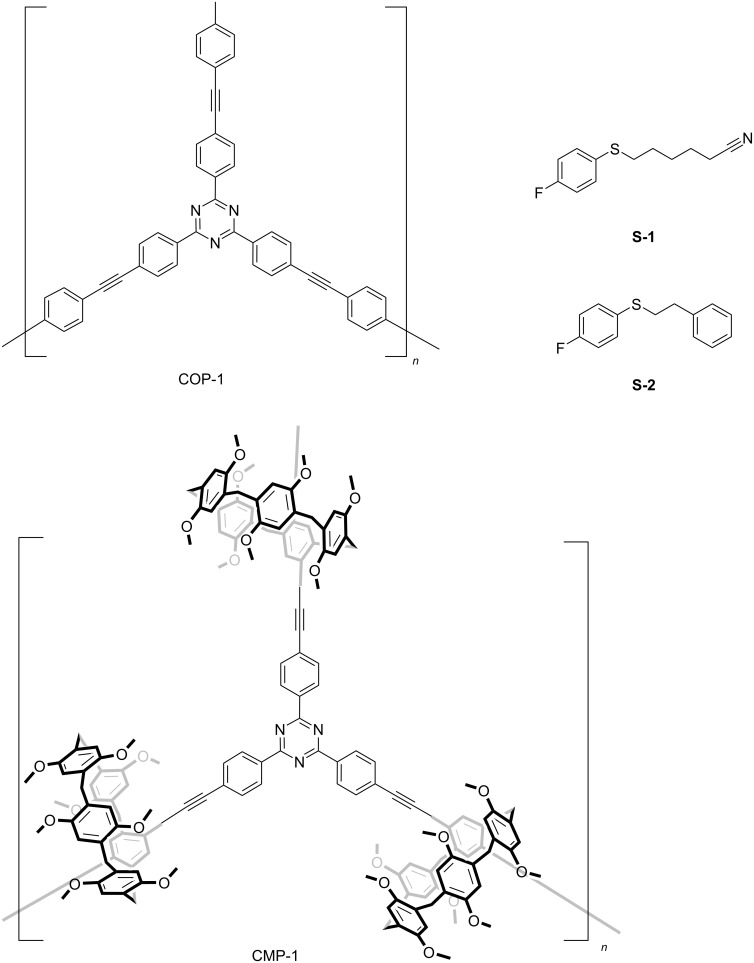
Structures of COP-1, CMP-1, and their substrate **S-1** and **S-2**.

Wen and co-workers reported the selective photocatalytic oxidation of sulfides in the presence of conjugated macrocycle polymers (COP) with pillar[5]arene struts (Figure 17) [55]. The host–guest interactions between pillar[5]arene and the substrates were confirmed as the main cause of the difference. The guest-like substrate **S-1** exhibited an enhanced oxidation efficiency over the non-guest-like substrate **S-2** under light-irradiation. In the presence of pillar[5]arene motifs, it tends to bind guest-like sulfide substrates and enhances the approximation of the activated oxygen species to the bonded sulfides. Consequently, the oxidation rate of **S-1** is accelerated. In the system COP-1, however, no selective binding exists, so that the activity towards both **S-1** and **S-2** was similar.

**Figure 18:**
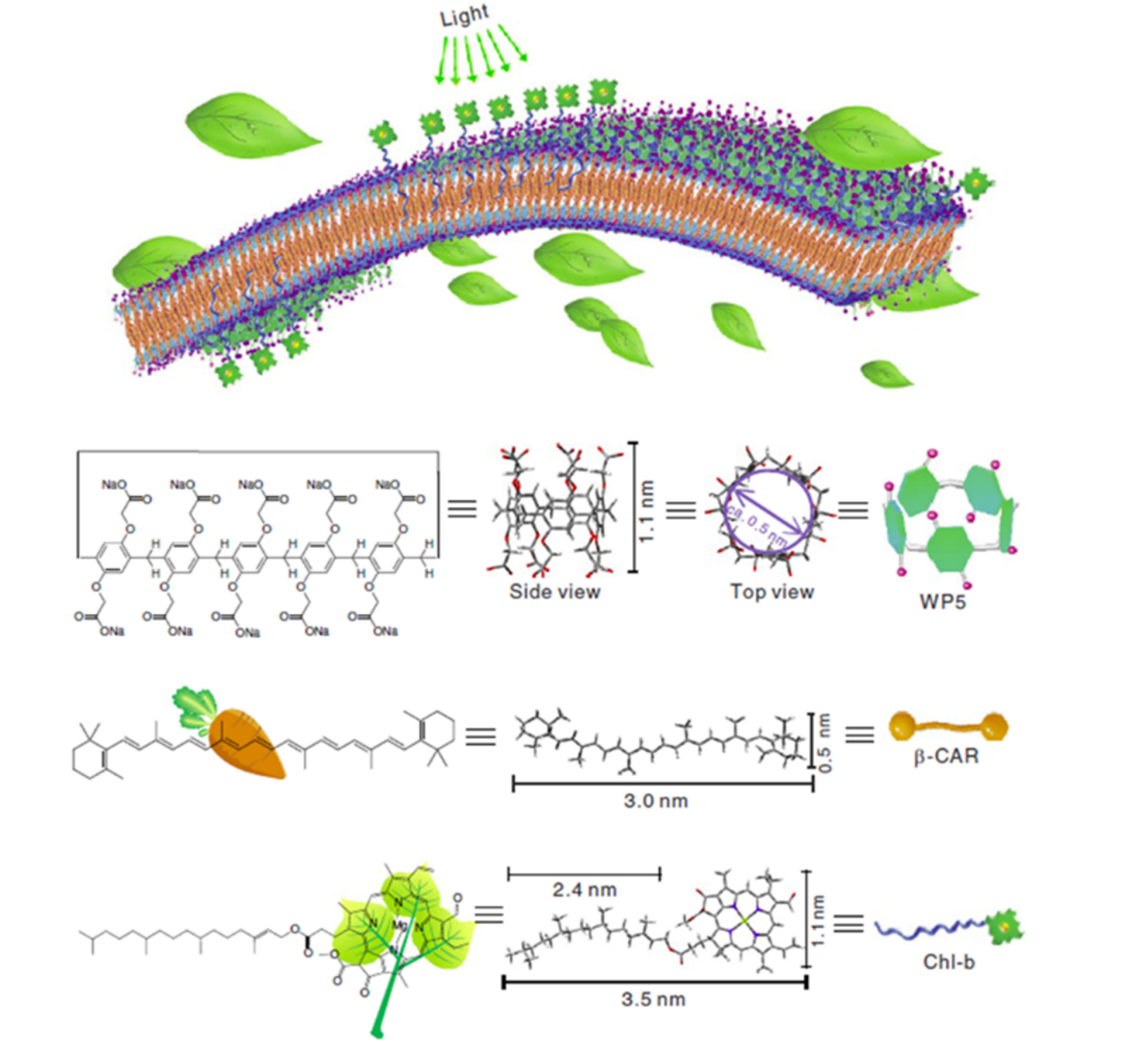
Supramolecular self-assembly of the light-harvesting system formed by WP5, β-CAR, and Chl-b. Reproduced from [56] (© 2016 Guowang Diao et al., distributed under the terms of the Creative Commons Attribution 4.0 International License, https://creativecommons.org/licenses/by/4.0).

Diao and co-workers reported a light-harvesting system for photocatalysis based on the water-soluble pillar[5]arene WP5 [56]. The large cavity volume of the formed nanoparticles enabled two guest β-carotene (β-CAR) and chlorophyll-b (Chl-b) molecules to be encapsulated within the cavity (Figure 18). The β-CAR and Chl-b molecules can absorb light and transfer energy, making the assemblies an efficient light-harvesting system. The host–guest binding stoichiometry is 1:1, and the association constant (*K*_a_) of WP5
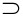
β-CAR was calculated to be (2.34 ± 2.06) × 10^5^ M^−1^. This system can function as a photocatalyst for the reduction of 4-nitrophenol after irradiation with a mercury lamp. The Pt or Ag nanoparticles could also be achieved by the photocatalyst property of the system in the presence of ascorbic acid. The mechanism can be explained in a way where Chl-b firstly absorbs light and generates the excited state, followed by the reduction through ascorbic acid. Then, the resulting Chl-b radical anion could catalyze the reduction of the respective metal.

**Figure 19:**
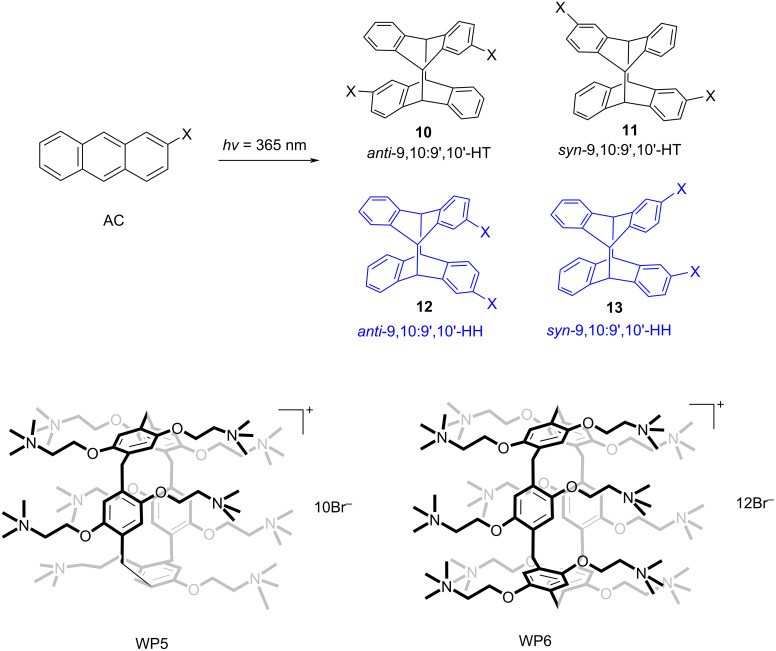
Photocyclodimerization of AC based on WP5 and WP6.

Photocatalysis with pillararenes often makes use of host-induced self-assemblies. However, their hydrophobic cavity may also act as a confined nanospace for photocatalysis [57]. Yang and co-workers reported the photocyclodimerization of AC with the water-soluble pillar[6]arene WP6 (Figure 19). The HH/HT yield ratio of the HH- and HT-photodimers is 0.75 in the presence of WP6, which is greatly improved compared to that without WP6 (HH/HT 0.30). This phenomenon may be due to the reduced electrostatic repulsion between the carboxylate groups of AC and the cationic ammonium groups of WP6. Although the selectivity is enhanced, the reaction rate constant of AC with WP6 is 4 times slower compared to that without any host molecule, which may be due to the mismatching between the photoreactive positions of AC and the cavity of WP6. Consequently, the 1:2 complex could inhibit the reaction.

## Conclusion

In summary, this minireview highlighted some widely used macrocycle-based photocatalytic systems, CEs, CDs, CBs, CAs, and PAs, for various photocatalytic reactions. The role of the photochemical macrocyclic-host-assisted catalytic reaction of different guests as well as hybrid materials in energy transfer were summarized. Moreover, the mechanisms of these supramolecular systems during the photochemical reactions were also highlighted. Due to the inherent limiting cavity and strong bonding ability with various guests, macrocycles function as a tool to promote the specific conversion of substrates. On the one hand, the close inclusion of substrates by macrocycles can realize the intervention in, and regulation of the reaction, and thereby developing a new type of catalytic system. On the other hand, macrocycles also provide a close space between the energy donors and acceptors, resulting in a highly efficient energy transfer to achieve certain reactions.

Although macrocycle-based catalysis has developed into a general catalytic strategy and has widely been used in various photocatalytic reactions, there are still several issues to be addressed: 1) To develop supramolecular photocatalytic systems, it is critical to have a thorough understanding of the photochemical and photophysical aspects of the reactions, such as the kinetic equilibrium constants, the reaction velocity, and the quantum yield. Therefore, a new criterion needs to be investigated for the physical parameters of these specific reactions. 2) We also need to develop novel macrocycles with chromophores. Most of the current macrocycles only provide the suitable cavities for the reactions, however, if a macrocycle has inherent chromophores that can function as photosensitizers, the efficiency of the energy transfer will be higher and the systems will be simplified. 3) Other kinds of macrocycle-based catalysis need to be explored, such as esterification, polymerization, redox reaction, etc. 4) Hyperbranched macrocycle-based polymeric nanocomposites can produce a better photocatalytic performance compared to the respective monomers and linear analogs due to their more accessible catalytic sites, whereas comparative studies are very limited. In addition, hyperbranched macrocycle-functionalized polymers can act as a stabilizer to control the size and distribution of the NPs and are able to intensely regulate the photocatalytic performance. 5) To improve the sustainability, noble metals need to be replaced by earth-abundant metals. However, in most cases, earth-abundant metals have a lower photocatalytic activity compared to noble metals in supramolecular materials. Therefore, there is an urgent demand to search for earth-abundant metals with a high activity. In conclusion, investigations on macrocycle-based catalysis are still needed to effectively extend their potential applications to solve chemical issues in industrial engineering.

## References

[1] Bornscheuer U T (2016). Nature.

[2] Breslow R (1995). Acc Chem Res.

[3] Raynal M, Ballester P, Vidal-Ferran A, van Leeuwen P W N M (2014). Chem Soc Rev.

[4] Pemberton B C, Raghunathan R, Volla S, Sivaguru J (2012). Chem – Eur J.

[5] Yang C, Inoue Y (2014). Chem Soc Rev.

[6] Brimioulle R, Lenhart D, Maturi M M, Bach T (2015). Angew Chem, Int Ed.

[7] Vallavoju N, Sivaguru J (2014). Chem Soc Rev.

[8] You L, Zha D, Anslyn E V (2015). Chem Rev.

[9] Poplata S, Tröster A, Zou Y-Q, Bach T (2016). Chem Rev.

[10] Chen Z, Lohr A, Saha-Möller C R, Würthner F (2009). Chem Soc Rev.

[11] Li F, Gorle A K, Ranson M, Vine K L, Kinobe R, Feterl M, Warner J M, Keene F R, Collins J G, Day A I (2017). Org Biomol Chem.

[12] Tang H, Sutherland A S M, Osusky L M, Li Y, Holzwarth J F, Bohne C (2014). Photochem Photobiol Sci.

[13] Pisani M J, Zhao Y, Wallace L, Woodward C E, Keene F R, Day A I, Collins J G (2010). Dalton Trans.

[14] Cullen W, Misuraca M C, Hunter C A, Williams N H, Ward M D (2016). Nat Chem.

[15] Yoshizawa M, Tamura M, Fujita M (2006). Science.

[16] Steed J W (2001). Coord Chem Rev.

[17] Gatto V J, Miller S R, Gokel G W (1990). Org Synth.

[18] Fedorova O, Fedorov Y V, Gulakova E, Schepel N, Alfimov M, Goli U, Saltiel J (2007). Photochem Photobiol Sci.

[19] Maligaspe E, Tkachenko N V, Subbaiyan N K, Chitta R, Zandler M E, Lemmetyinen H, D’Souza F (2009). J Phys Chem A.

[20] Cibulka R, Vasold R, König B (2004). Chem – Eur J.

[21] Szejtli J (1998). Chem Rev.

[22] Chen Y, Liu Y (2010). Chem Soc Rev.

[23] Wang J, Chen Y, Cheng N, Feng L, Gu B-H, Liu Y (2019). ACS Appl Bio Mater.

[24] Inoue Y, Wada T, Sugahara N, Yamamoto K, Kimura K, Tong L-H, Gao X-M, Hou Z-J, Liu Y (2000). J Org Chem.

[25] Wei X, Wu W, Matsushita R, Yan Z, Zhou D, Chruma J J, Nishijima M, Fukuhara G, Mori T, Inoue Y (2018). J Am Chem Soc.

[26] Yi J, Liang W, Wei X, Yao J, Yan Z, Su D, Zhong Z, Gao G, Wu W, Yang C (2018). Chin Chem Lett.

[27] Ji J, Wu W, Liang W, Cheng G, Matsushita R, Yan Z, Wei X, Rao M, Yuan D-Q, Fukuhara G (2019). J Am Chem Soc.

[28] Zhu H, Goswami N, Yao Q, Chen T, Liu Y, Xu Q, Chen D, Lu J, Xie J (2018). J Mater Chem A.

[29] Wang J, Feng Y-X, Zhang M, Zhang C, Li M, Li S-J, Zhang W, Lu T-B (2020). CCS Chem.

[30] Kato M, Kon K, Hirayama J, Yagi I (2019). New J Chem.

[31] Kumar R, Lee Y O, Bhalla V, Kumar M, Kim J S (2014). Chem Soc Rev.

[32] Dondoni A, Marra A (2010). Chem Rev.

[33] Huang J-F, Liu J-M, Xiao L-M, Zhong Y-H, Liu L, Qin S, Guo J, Su C-Y (2019). J Mater Chem A.

[34] Shalaeva Y V, Morozova J E, Gubaidullin A T, Saifina A F, Shumatbaeva A M, Nizameev I R, Kadirov M K, Ovsyannikov A S, Antipin I S (2020). Colloids Surf, A.

[35] Yang X-X, Yu W-D, Yi X-Y, Liu C (2020). Inorg Chem.

[36] Chen Y-F, Huang J-F, Shen M-H, Liu J-M, Huang L-B, Zhong Y-H, Qin S, Guo J, Su C-Y (2019). J Mater Chem A.

[37] Wang X, Yu Y, Wang Z, Zheng J, Bi Y, Zheng Z (2020). Inorg Chem.

[38] Zhou R, Srinivasan M P (2016). Mater Today Chem.

[39] Freeman W A, Mock W L, Shih N Y (1981). J Am Chem Soc.

[40] Day A I, Blanch R J, Arnold A P, Lorenzo S, Lewis G R, Dance I (2002). Angew Chem, Int Ed.

[41] Lee J W, Samal S, Selvapalam N, Kim H-J, Kim K (2003). Acc Chem Res.

[42] Tang X, Huang Z, Chen H, Kang Y, Xu J-F, Zhang X (2018). Angew Chem, Int Ed.

[43] Lei L, Luo L, Wu X-L, Liao G-H, Wu L-Z, Tung C-H (2008). Tetrahedron Lett.

[44] Biedermann F, Ross I, Scherman O A (2014). Polym Chem.

[45] Smitka J, Lemos A, Porel M, Jockusch S, Belderrain T R, Tesařová E, Da Silva J P (2014). Photochem Photobiol Sci.

[46] Hu X, Liu F, Zhang X, Zhao Z, Liu S (2020). Chem Sci.

[47] Pemberton B C, Barooah N, Srivatsava D K, Sivaguru J (2010). Chem Commun.

[48] Yang Y, An Z (2019). Polym Chem.

[49] Song D, Li B, Li X, Sun X, Li J, Li C, Xu T, Zhu Y, Li F, Wang N (2020). ChemSusChem.

[50] Ogoshi T, Kanai S, Fujinami S, Yamagishi T-a, Nakamoto Y (2008). J Am Chem Soc.

[51] Xue M, Yang Y, Chi X, Zhang Z, Huang F (2012). Acc Chem Res.

[52] Wang K, Jordan J H, Velmurugan K, Tian X, Zuo M, Hu X-Y, Wang L (2021). Angew Chem, Int Ed.

[53] Zuo M, Qian W, Hao M, Wang K, Hu X-Y, Wang L (2021). Chin Chem Lett.

[54] Hao M, Sun G, Zuo M, Xu Z, Chen Y, Hu X-Y, Wang L (2020). Angew Chem.

[55] Qiang H, Chen T, Wang Z, Li W, Guo Y, Yang J, Jia X, Yang H, Hu W, Wen K (2020). Chin Chem Lett.

[56] Sun Y, Guo F, Zuo T, Hua J, Diao G (2016). Nat Commun.

[57] Gui J-C, Yan Z-Q, Peng Y, Yi J-G, Zhou D-Y, Su D, Zhong Z-H, Gao G-W, Wu W-H, Yang C (2016). Chin Chem Lett.

